# Mendel’s controlled pollination experiments in *Mirabilis jalapa* confirmed his discovery of the gamete theory of inheritance in *Pisum*

**DOI:** 10.1186/s41065-022-00232-1

**Published:** 2022-03-26

**Authors:** Hui Zhang, Xiaoxi Zhao, Fan Zhao, Jianshan Han, Kun Sun

**Affiliations:** College of Life Science, Northwest Normal University, 967 Anning East Road, Lanzhou, 730070 China

**Keywords:** *Mirabilis jalapa*, Controlled pollination, Gamete theory of inheritance, Hereditary factor, Variable hybrids, Constant hybrids

## Abstract

The historian studies revealed during Mendel’s later research period when mainly focusing on the constant hybrid in *Hieracium*, he had to be intervened to conduct the controlled pollination experiments in *Mirabilis jalapa*. Two letters to Nageli recorded the experimental aim was to disprove Darwin’s opinion regarding three pollen grains required for one fertilization (note: that could completely destroy his previous discovery of segregation inheritance in variable hybrid in *Pisum*, for it was expressed in a mathematical equation). The experimental results of single pollen grain pollination confirmed the referenced view of one pollen cell uniting one egg cell in plant fertilization; the further pedigree introduction of the single and of the designed two pollen grain experiment succeeded in exemplifying that one hereditary factor carried by one gamete (pollen cell or egg cell) can independently transmit a trait to offspring. Here we coined the observation as the Gamete Theory of Inheritance. Remarkably, in contrast with the bulked pollination experiment, in this system, Mendel could easily manipulate a hereditary factor by merely taking a gamete as a carrier. Then, Mendel’s work in *M. jalapa* together with the previous *Pisum* study was able to jointly suppport his second lecture content that regarded “gamete formation, fertilization, and seed development” and also regarded hereditary factors in the processes. All in all, the 1866 paper was published during a rapid burst of interest in hybrid species likely induced by Darwin, and Mendel’s attempts at accommodation of the two incompatible inheritances of segregation in variable hybrids versus of nonsegregation in constant hybrids might be responsible for some historical controversies when understanding his discovery of inheritance.

**Supplementary Information:**

The online version contains supplementary material available at 10.1186/s41065-022-00232-1.

Recently, more and more historical studies have revealed that Mendel had a broad scope of research interests in genetics that were connected with both variable and constant hybrids [1–3]. As a diligent experimenter of plant hybridization, Mendel’s most significant achievement was in his first research period from 1856 to 1863 in which he discovered the law of inheritance regarding variable hybrids in his *Pisum* experiments (in the 2th letter to Nageli) [4, 5]. But then, he did shift his research topics at certain important moment in his life [1, 3]. During 1866, Mendel was publishing his famous paper and in parallel, initiating his main investigation of constant hybrids in *Hieracium* [3]. Here, we present a reconstruction of Mendel’s controlled pollination experiments in *Mirabilis jalapa* that was later conducted in his second research period on *Hieracium* (the 8th and 9th letter to Nageli) [5]. Superior to traditional bulked pollination, the methods of this experiment provided a platform for Mendel to concentrate on one pollen grain and one ovule, both of which were assumed to be respectively equipped with hereditary factors. As a continuous study of variable hybrids, Mendel’s *M. jalapa* work merits further exploration as an indispensable part of his inheritance investigation, especially against the background of his interest in constant hybrids already overshadowing that in variable hybrids.

## Mendel’s discovery of inheritance and its oral presentation

In Brno, Mendel was recognized as a famous horticulturist and breeder by his contemporaries, and his interest in inheritance may have developed from the local cross-breeding tradition and his plant improvement practices [6–8]. As he claimed, “to observe these changes for two differing traits (in *Pisum*) respectively, and to investigate the law according to which they occur in the successive generations, was the task of the experiment” (Mendel’s 1866 paper was in German; also see [9] in both German and English that was translated by S. Müller-Wille and K. Hall, available at: http://www.bshs.org.uk/bshs-translations /mendel). Consequently, he achieved the discovery of segregation and other inheritance principles. Then, he continued to undertake “a number of hybridizations in 1863 and 1864” to find suitable plants for an extended series of experiments (the 2nd letter) [5]. Besides the case of *Phaseolus* likely orally presented at the 1865 meeting and written in the 1866 paper, Mendel also shared his work on crosses between *Verbascum* and *Campanula* species at the June 1865 meeting of the Natural Science Society of Brno (NSSB) [10]. In light of the experiments Mendel actually conducted, it is concluded that he still investigated the inheritance and evolution of variable hybrids in 1864 and 1865, despite of claims that Mendel had read the German edition of *On the Origin of Species* at that time [11]. In February and March of 1865, Mendel intentionally divided his work into two lectures at the monthly NSSB meeting, one on species evolution of and another on inheritance of variable hybrids, and he deliberately reported his discovery of inheritance in *Pisum* at the second lecture in particular [12].

Fortunately, there are many surviving reports about Mendel’s second lecture in local newspapers that have become newly accessible. In *Neuigkeiten*, it was recorded, Mendel declared that he had made the discovery “about (reproductive) cell formation, fertilization, and seed development in general and in the case of hybrids in particular,…...” After Mendel’s report, “von Niessl added ……that with the aid of the microscope he had observed hybridizations in fungi, mosses and algae, and that further observations of this kind not only supported existing hypotheses but will also give further interesting clarifications” [13, 14]. In *Mährischer Korrespondent*, Mendel’s discovery was reported as of “cell and the reproduction of the plants by fertilization” [8]. *Brünner Zeitung* reported it as follows: “After a clear presentation of the most recent findings of the research on the genesis and development of the plant germ in general, the lecturer sought to utilize them in the formation of the hybrids, and *developed a hypothesis concerning the factors involved in this process*”(emphasis from the cited author) [14]. From these quotes, an overview becomes clear. In his second lecture, Mendel orally presented his discovery of inheritance in variable hybrids as the hypothesis of hereditary factors regarding plant sexual reproduction in general, but especially in hybrid formation, that contains three successive processes: gamete formation, fertilization, and seed development. The microscopic basis of hereditary factors in hybridization was discussed after the report, and all agreed to need further cytological observation to clarify it. Indeed, the words in *Brünner Zeitung* contained the sole record in history on Mendel’s open announcement of his discovery of hereditary factor. Here, we report that Mendel’s controlled pollination experiments in *Mirabilis jalapa* really comprised the pretty study that could perfectly confirm his hereditary factor hypothesis, which will be deeply and broadly presented in the later section. Sure, if there were not the surviving letters that contained Mendel’s introduction to Nageli about his *M. jalapa* study, we would not clearly understand Mendel’s hypothesis regarding hereditary factors, because Mendel had fused it with other writing themes when he published the *Pisum* work in 1866.

## Publication of the full breadth of Mendel’s genetics discoveries

Since the beginning of the scientific activity of NSSB that independently run at the end of 1861, “its members, in the spirit of the time, showed great interest in plant hybrids,” e.g., natural hybrid species in *Cirsium*, *Hieracium*, and *Verbascum* were separately discussed one after another at the society sessions [10]. Mendel considered “those hybrids which are collected in the wild can be used as secondary evidence only, as long as their origin is not unequivocally known” (the 1st letter) [5]. His research interest was being attracted by artificial constant hybrids that were recorded in Gärtner’s *Versuche und Beobachtungen über die Bastarderzeugung im Pflanzenreich* for their constant effects in reproduction just as those observed in stable species [2]. As for the natural intermediate forms in *Hieracium*, whether they belong to hybrid species or transient forms in the process of speciation was still an open question at the time. Thus, as soon as the true breeding lines were established in 1865, Mendel immediately launched his crossing experiments in *Hieracium*, as well as in *Cirsium*, and *Geum* to mainly explore speciation through formation of constant hybrids in 1866 (the 1st letter) [3, 5]. These works also occurred during the time he was preparing the paper for publication just after his two speeches in 1865.

No doubt, Mendel was able to confidently present his hypothesis of hereditary factors discovered in *Pisum*, even under such an atmosphere, because at the meeting what he reported should be experiments he had actually conducted before, certainly, almost all work regarding variable hybrids. However, “when he was preparing his paper……Darwin’s writings influenced Mendel’s interpretations and theory” [15]. He intended to take “the spirit of the time” into account so that both variable and constant hybrids were considered in the publication with respect to inheritance and species evolution [9]. Regardless of the respect of species evolution, here we can image, when Mendel tried to accommodate the inheritance mechanism of segregation in variable hybrids along with of non-segregation in constant hybrids in the paper, difficulty emerged. As later we know, here is a huge biological difference between sexual and nonsexual reproduction (i.e., apomixis). Similar to the historical battle between particle inheritance and blending inheritance [16], Mendel’s obtained findings in variable hybrids in *Pisum* were rather incompatible with his only conjecture that would be used to guide his subsequent study of constant hybrids in *Hieracium*. Indeed, he also repeatedly expressed that the two cases were essentially different in the 1866 paper [9]. And, the two incompatible mechanisms were so mutually inconsistent that they could be easily separated and demonstrated respectively (Fig. 2 in van Dijk and Ellis) [3].

In order to compromise his thoughts about constant hybrids, Mendel’s finding of inheritance in *Pisum* was broadly discounted in his paper. In particular, the so-called particle inheritance was extremely diminished to accommodate the coexistence of blending inheritance [9]. Mendel even resorted to presenting an incorrect view that only unlike elements are mutually exclusive but similar elements appear not to be, as has already been noted by Olby [17]. Despite the terms “Factor” and “Element” (“Factoren” and “Elementen” in plural in the original German text) as well as their synonyms being dispersed throughout the context, regretfully, there was no unambiguous presentation of such a theory concerning hereditary factors as he had orally presented just one year before. Even the already demonstrated and now well-accepted laws of segregation and of free combination were only parceled together and finally reported as a part of a whole hypothesis as follows,“the reduction attempted here of the essential difference in the development of hybrids to a lasting or passing association of differing cell elements can of course only claim the value of a hypothesis for which further scope remains open due to the lack of firm data” [9].

Consequently, because Mendel’s notebook containing the unique record of his experiments in *Pisum* was destroyed, the paper has brought out many controversies in history [18]. For example, Mendel has been argued to be non-Mendelian and even to not hold a theory of the gene [17]. Mayr further argued that “Mendel did not, by a single stroke, create the whole modern theory of genetics” [19]. Here, we note that Mendel’s controlled pollination experiments in *M. jalapa* explicitly corroborated his hypothesis of hereditary factors previously obtained from the work in *Pisum* and also provides adequate support to resolve Olby and Mayr’s critiques.

## Deficiencies of Mendel’s experiments in *Pisum*

Indeed, even if the impact from his later emerged interest in *Hieracium* was gotten rid off, there were still some deficiencies in Mendel’s *Pisum* study presented in 1866 paper. In response to the audience’s inquiry about the cytological characteristics of the hereditary factor during the meeting, Mendel also pointed out the deficiency of his findings in the paper, “for instance, relating to the composition of the hybrid fertilizing cells”. Regarding male and female reproductive cells, Mendel first stated that “both are equipped with the material for creating complete identical individuals” [20, 21], implying that Mendel actually possessed three research targets: internal factors, gametes, and external traits. However, at that time, except for external traits, “none of these (gametes and factors) was concerned with directly observable things”, so what Mendel could do was “to make predictions as to the outcome of the further experiments” [22]. Even in his *M. jalapa* study reported here, Mendel also adopted a similar method to verify his assumption. That is, marking pollen cells and egg cells with the external traits of the plants initially bearing them, conducting pollinations, and finally checking the predicted ratios of the observable traits in the progeny. To some extent, this also brought about an incorrect impression that Mendel was only an empirical scientist more interested in mathematical laws than in hereditary factors themselves. Indeed, the law of segregation and of free combination were respectively testified through his obtaining the predicted 1:1 and 1:1:1:1 ratios of various kinds of traits (*A*:*a*) or trait combinations (*AB*:*Ab*:*aB*:*ab*) in testcross experiments. That is also why the two laws became well accepted and are taught in textbooks.

Except the limitations due to contemporary condition metioned above, the most substantial criticism was “Mendel himself never says explicitly that they (“similar trait”, “gleichartigen Merkmale” in German) must be represented in the gamete by only a single element” [19]. The testcrosses were designed to only detect the inner composition of gametes, so the collectivity of a group of pollen grains in the bulked pollinations was enough to meet this need. The normal behaviors of hereditary factors in gamete formation could be inferred from the upstream process of plant sex reproduction. However, what were the relationships among the three research targets? In particular, what was the behavior of one gamete carrying one factor in the midstream process of fertilization, as well as the function of one factor for determining one trait in the downstream processes of seed development? All of these research focuses were treated just as a series of black boxes with no answer in *Pisum*. As for fertilization, Mendel only stated that “according to the opinion of famous physiologists, in phanerogams one germ and one pollen cell respectively unite to form a single cell”. As for plant development from primordia cell, Mendel assumed “this development occurs according to a constant law, which is grounded in the material constitution and arrangement of the elements that attained a viable union in the cell” [9]. After introducing the assumed agreement in development of homozygous progeny with of the mother plant, Mendel divided the development of heterozygous progeny into two types. One was variable hybrids in which the two conflicting elements obey the laws of segregation and the other laws, like in *Pisum*; the other was constant hybrids, where the compromise of dissimilar elements was assumed to be entire and permanent, however, without any experimental evidence [9].

All in all, under such a background almost all concerning hybrid species, Mendel was so eager to conclude his *Pisum* study and merely wanted to simply check its general applicability in other plants, and thus without hesitation he shifted to hybridization experiments in *Hieracium* and so on. He not only cut the findings in variable hybrids to accommodate his view regarding constant hybrids, but also left his hypothesis of hereditary factors in the two remaining respects, i.e., fertilization and seed development, unverified. Indeed, he did not change his intentions until 2 years later when reading Darwin’s citation of Naudin’s experiment in *M. jalapa*.

## Darwin’s citation of Naudin’s experiments in *M. jalapa*

**Fig. 1:**
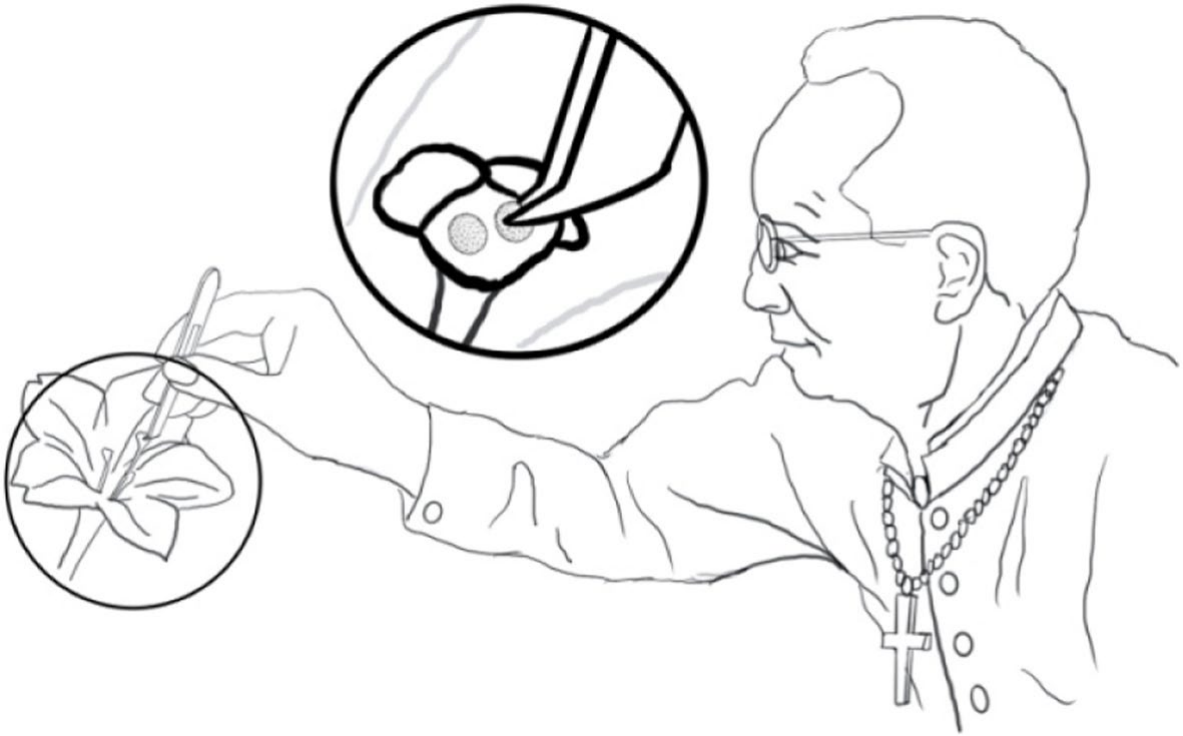
The controlled pollination experiment conducted in *Mirabilis jalapa* by Mendel

Mendel’s attention to Darwin was always lasting in his research career. In 1868, Mendel read *The Variation of Animals and Plants under Domestication* that was published in the same year. In Chapter 27, on “Provisional hypothesis of pangenesis”, Darwin divided organism reproduction into sexual and asexual types. When comparing the two kinds of reproduction, Darwin cited studies conducted in the animals *Teredo* and batrachians, claiming that more than one sperm would be necessary to fertilize a single egg, and for certain plant species in the genus *Malva*, even 40 pollen grains only yielded several small seeds [23]. Then, Darwin introduced in detail the pollination experiments in *Mirabilis jalapa* conducted by the French botanist Charles Naudin: “In the case of *Mirabilis* the pollen grains are extraordinarily large, and the ovarium contains only a single ovule; and these circumstances led Naudin to make the following experiments: a flower was fertilized by three grains and succeeded perfectly; twelve flowers were fertilised by two grains, and seventeen flowers by a single grain, and of these one flower alone in each lot perfected its seed: and it deserves especial notice that the plants produced by these two seeds never attained their proper dimensions, and bore flowers of remarkably small size.” In the same chapter, Darwin continued that “the ovules and the male element have equal power of transmitting every single character”, but “from unknown causes, one sex sometimes has a much stronger power of transmission than the other” [23]. Here, Darwin’s claim differed strongly from Mendel’s discovery of equal contributions of both parents to their offspring. In particular, the erroneous citation of three pollen grains required to fertilize one egg thoroughly destroyed the biological basis of his discovery of hereditary factors. Mendel then immediately began to repeat Naudin’s experiments in *M. jalapa* (Fig. 1) in the same year, i.e., 1868, and also communicated his experimental findings to Nageli [4, 5].

## Two letters to Nageli

1. ***Mendel’s 8th letter to Nageli, Brno, July 3, 1870***

“Because of my eye ailment I was not able to start any other hybridization experiments last year. But one experiment seemed to me to be so important that I could not bring myself to postpone it to some later date. It concerns the opinion of Naudin and Darwin that a single pollen grain does not suffice for fertilization of the ovule. I used *Mirabilis jalapa* for an experimental plant, as Naudin had done; the result of my experiment is, however, completely different. From fertilizations with single pollen grains, I obtained 18 well-developed seeds, and from these an equal number of plants, of which ten are already in bloom. The majority of the plants are just as vigorous as those derived from free self-fertilization. A few specimens are somewhat stunted thus far, but after the success of all the others, the cause must lie in the fact that not all pollen grains are equally capable of fertilization, and that furthermore, in the experiment mentioned, the competition of other pollen grains was excluded. When several are competing, we can probably assume that only the strongest ones succeed in effecting fertilization.

“Besides, I want to repeat the experiment; and it should also be possible to prove directly by experiment whether or not two or more pollen grains can participate simultaneously in the fertilization of the ovule in *Mirabilis*. According to Naudin, at least three are needed.”

2. ***Mendel’s 9th letter to Nageli, Brno, September 27, 1870***

“The experiment designed to solve the question whether or not a single pollen grain suffices for fertilization, was repeated with *Mirabilis Jalapa,* with the same results as last year. Plants obtained from last year’s fertilizations using a single pollen grain cannot be distinguished in any way from those produced by self-fertilization. In the beginning it seemed as if individual plants might lag behind in development; later, however, they completely made up the loss.

“Under way is another experiment with *Mirabilis*, designed to find out also whether two pollen grains may simultaneously participate in fertilization. The varieties with crimson red, yellow, and white, flowers, respectively are constant when raised from seed, as I know from experience, and the hybrids which first result from the crosses crimson + yellow and crimson + white show no variations in their characteristic coloration. Both fertilizations succeed equally well and thus no difference in the degree of relationship [among the three varieties] is apparent. In the crimson variety a fairly large number of fertilizations were undertaken in such a way that two pollen grains were simultaneously put on each stigma, one of the yellow, and one of the white variety. Since the resultant flower colors of the crosses crimson + yellow and crimson + white are known, it will be shown next year whether in addition to the hybrid colors still a third color will appear, one explainable by joint action of the two pollen grains.

“In the latter case, development of the progeny should also be different from that in the two simple color hybrids. These behave like *Pisum*, and half of the first generation again produces the hybrid color, while the other half receives the two parental colors in equal parts, and remains constant in the next generation. Those offspring of the hybrid crimson + yellow, which received the parental colors in the first generation, have also proved themselves to be constant as regards color in the second generation raised from seeds. Both colors reappear in pure form, as though they had never been in hybrid combination. Darwin and Virchow have pointed to the high degree of independence that is typical for individual characters and whole groups of characters in animals and plants. The behavior of plant hybrids indisputably furnishes an important proof of the correctness of this point of view.”

## Reconstruction of Mendel’s experiment in *M. jalapa*

*M. jalapa* L., commonly known as Four O’clock, is an ornamental flower that originated in Peru and was introduced to Europe in 1525. *M. jalapa* is self-compatible and has flower colors of crimson, yellow, dominant white, recessive white, among others. Since the eighteenth century, *M. jalapa* has been used as an experimental species by pioneering hybridists, and was also used later by Naudin and Carl Correns, among others [24]. To disprove Darwin’s citation of Naudin’s observation, in 1868, Mendel collected (or already possessed) copious materials of the genus *Mirabilis*, including several cultivars of *M. jalapa* bearing various flowers, which he used to conduct controlled pollination experiments. Table 1 listed all hybridization experiments that Mendel executed in *Mirabilis* as they were introduced or mentioned in his letters.

**Table 1:** Mendel’s hybridization experiments in *Mirabilis*

Pollination type		Combination/ Year		1868^a^	1869	1870
Bulked pollination		*M. jalapa* × *M. longiflora*		C	F_1_	F_2_
		*M.jalapa*^b^	crimson × yellow	C	F_1_	F_2_
			crimson × white	C	F_1_	F_2_
Controlled pollination	Single pollen grain^c^	*M. jalapa*	crimson × yellow	C	F_1_/C	F_2_/F_1_/C
			crimson × white	C	F_1_/C	F_2_/F_1_/C
	Two pollen grains	*M. jalapa*	crimson × (yellow + white)			C

^a^ as the author of Mendel’s biography [1], Orel cited Cetl’s paper in *Folia biologia* [25] where the start time of Mendel’s experiment in *Mirabilis* was listed as 1867; however, there was no direct evidence for this date; ^b^ Mendel might have executed the method of bulked pollination as listed, but the probability of his conduction of their reciprocal crosses can not be excluded; ^c^ the two combinations of the single pollen grain experiments were repeatedly conducted over 3 years, showing Mendel’s rigorousness and the difficulties associated with achieving successful operation

Several things are worth noting: 1) in his seventh letter to Nageli (April 15, 1869), Mendel stated “I have one specimen of the interesting hybrid *M. jalapa* × *M. longiflora.* A few plants were obtained from the small number of seeds which it bore last summer; they are, however, still too delicate to stand transportation” (in the 7th letter) [5]. The bulked pollination of inter-specific hybridization experiment was consistent with his enduring communication with Nageli on the hybridization of *Hieracium*. And, almost all of Mendel’s experiments in *Mirabilis* had been spontaneously initiated in 1868, most likely because of Darwin’s direct influence; 2) in single pollen grain pollination experiments, the probability of Mendel’s conduction of selfing and other hybridization combinations by using the same materials cannot be excluded; however, the most fruitful operation included annual pollinations of crimson × yellow and crimson × white from 1868 to 1870, with the obtained results of F_2_ generation in crimson × yellow reported to Nageli; 3) Mendel utilized the normal method of bulked pollination with the same materials to conduct a positive control for his controlled pollination experiments. From this, he “experienced” that parental materials were constant (homozygous) and the intermediate flower coloration of the F_1_ hybrid showed no variations (incompletely dominance). His experience later inspired him to start his experiment on two pollen grains pollination in 1870; 4) Because of uncertain and surely regrettable reasons, Mendel’s letters containing resultant data of the two pollen grain experiments were lost, barely leaving the introduction of his initiation and detailed design of the experimental framework. For this very reason, it is necessary to reconstruct the pedigree framework of all of Mendel’s hybridization experiments in *M. jalapa*.

**Fig. 2:**
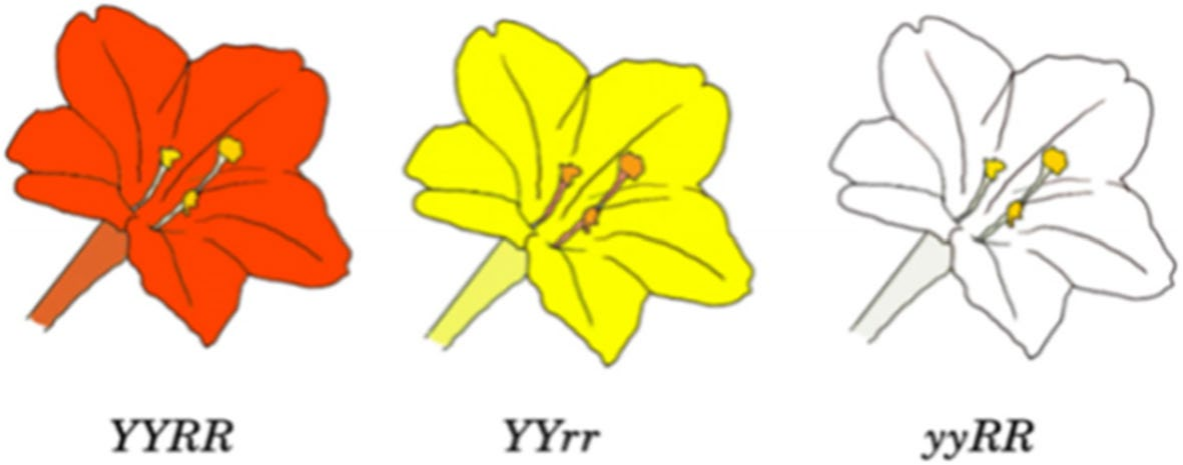
Phenotypes and inferred genotypes of Mendel’s Four O’clock materials

**Fig. 3:**
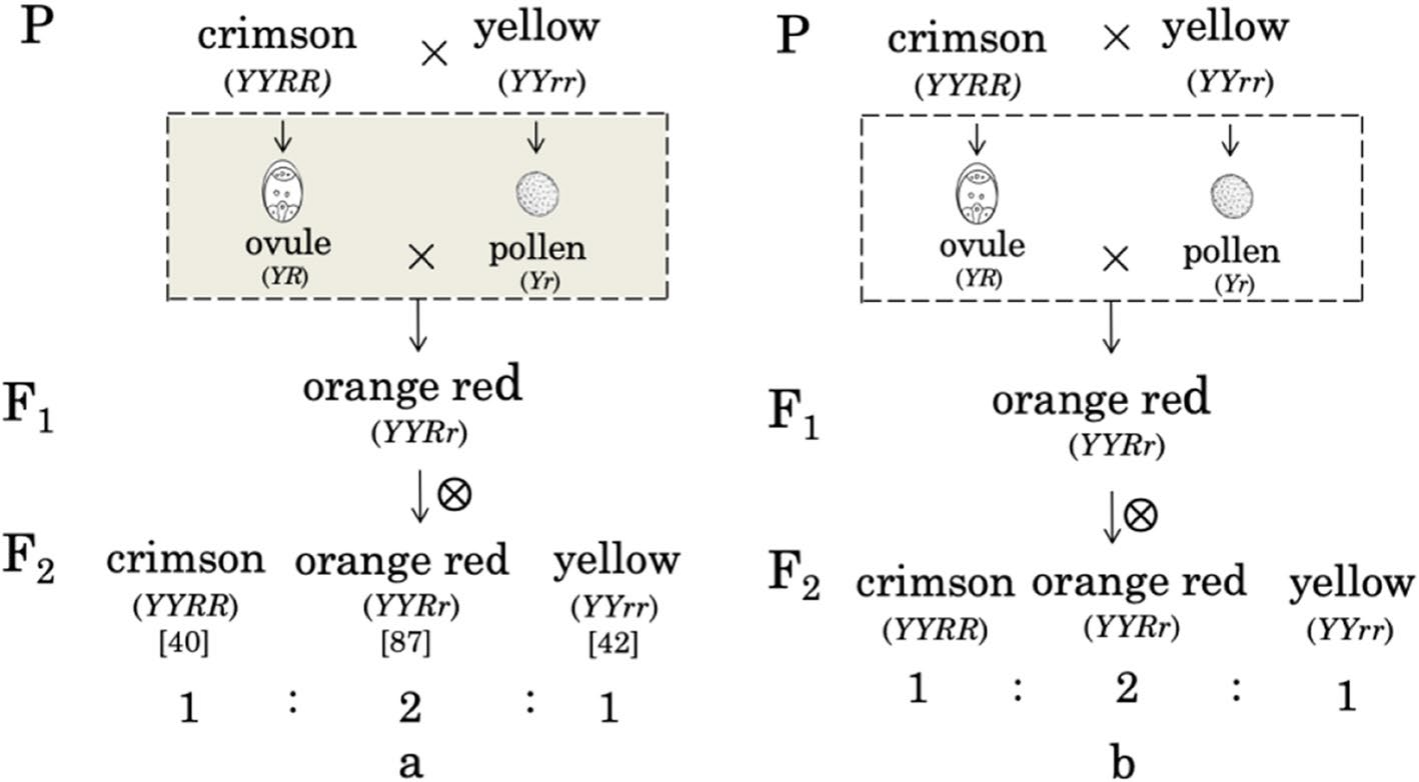
Putative pedigrees of Crimson × Yellow in *Mirabilis jalapa*. **a**, pedigree adopted from Showalter [27], the gray square indicates the imprecise relationship between pollens and eggs in bulked pollination before Mendel’s *M. jalapa* work; **b**. assumed pedigree of the single pollen experiment completed by Mendel; the rectangle marks Mendel’s precise operation between one pollen grain and one ovule in *M. jalapa*

All colored flower lines of *M. jalapa* were concluded to have derived from the crimson strain, the material basis of which was a mixture of magenta anthocyanin and a soluble yellow pigment [26]. The flower color in *M. jalapa* was considered as being controlled by two independent non-allelic loci with a certain kind of interaction: *Y* and *y* control the basic color yellow, and *R*, *Rp*, *rp*, and *r* are multiple alleles that modify *Y* and *y* to different degrees of red [27]. The genotypes of the crimson inbred lines may be *YYrr* or *YYRpRp*, those of the yellow inbred lines may be *YYrr* or *YYrprp*, and those of the dominant white varieties may be *yyRR* (recessive white *yyrr* was discovered in the twentieth century, and is therefore not considered here). It is known that *RP*, *Rp*, and *rp* in series of multiple *R* alleles show epistatic interaction in such a way that the white recessive character of *y* is covered up, leading to the segregation ratio of offspring deviating from 3:1 in general [24, 27]. Also, the F_1_ hybrid of Showalter’s crimson × yellow was stable orange red *YYRr*, while the F_1_ hybrid of the crimson × white was stable rhodamine purple *YyRR* [27]. Therefore, according to Mendel’s experience and the introduction above, it can be concluded that the genotypes of the materials used by Mendel might have been crimson *YYRR*, yellow *YYrr*, and white *yyRR*, respectively (Fig. 2). Then, Showalter’s hybridization pedigrees in crimson × yellow (Fig. 3a) and crimson × white (Fig. S1a) could be used to illustrate Mendel’s corresponding pedigrees of the same bulked-pollination experiments, with the gray square placed in the figures to highlight Mendel’s operational act in controlled pollination experiment in *M. jalapa*.

In the last step, an embryo sac and a pollen grain are drawn out to illustrate a female and a male gametophyte, visually highlighting a female and a male gamete. Considering the method of pedigree analysis that was created by Mendel himself, bisexual gametes are marked with a hereditary factor, as will be introduced in detail below. Thus, Mendel’s single pollen pollination pedigrees in crimson × yellow and crimson × white are demonstrated (Fig. 3b & S1b). The designed experimental framework of the two pollen grains experiment is also reconstructed (Fig. S2), based on Mendel’s introduction of his operations, which was partially finished in the summer of 1870, as well as his anticipation of future results. So far, Mendel’s experimental framework in *M. jalapa* has been recovered by using terms and symbols of modern genetics. Now, it is time to go back to Mendel’s time and evaluate the scientific content and academic significance of this framework in the field of Mendelian genetics.

## Evaluation of the experiment in *M. jalapa*

### Confirmation of one pollen cell uniting with one egg cell in fertilization

Mendel had discovered the law of inheritance by applying the method of bulked pollination in *Pisum*. Bateson ever complained that “the fact Mendel discovered …… is only elucidated as an *average* [emphasis by Bateson] result. Unfortunately …… even from the few recorded by Mendel himself, we see that the fluctuations are so great” [28]. Indeed, such argument was partially owing to the inevitably inaccuracy of statistical treatment commonly executed in all bulked pollination experiments. Once Mendel initiated his controlled pollination experiments in *M. jalapa*, the operating target was immediately changed from the bulk groups of pollen grains to only one or two pollen grain(s). As a result, the unavoidable fluctuation of resulting data in statistical analysis was altered into an oscillation of the success rate in multiple attempts of independent experiments. Each successful pollination operation, despite its difficulty, clearly produced an unquestionable result. Thus, the criticisms of Mendel’s work as “average” and “inexplicit” could be dispelled simply owing to the intrinsic accuracy of controlled pollination experiments in *M. jalapa*.

On the other hand, a recent study has identified a connection between the man Mendel called a “renowned physiologist” and Johann Nave, who might have shared Nathanael Pringsheim’s microscopic observation of the freshwater alga *Vaucheria sessilis*, in which one male gamete united with one female gamete in fertilization [14]. Dramatically, the knowledge gap between “a group of pollen grains” and “a pollen grain” early left in his *Pisum* study was unexpectedly challenged by Darwin’s citation of Naudin’s work. Thus, Mendel had to repeat Naudin’s experiment in *M. jalapa*. In essence, there is no difference between the two scientists’ single pollen grain pollination experiments. Naudin achieved a successful fertilization after 17 attempts, while Mendel achieved 18 successful pollinations followed by seed settings. Only, Mendel did not report how many pollination attempts he had made in 1868 and 1869. Thus, the experimental results of both scientists eventually confirmed the observation of the “renowned physiologist”. To a certain degree, Darwin’s opinion on biased contributions of both parents to offspring may be an erroneous inference from Naudin’s experiments, i.e., that the more pollen grains are used, the more successful fertilization is. Mendel scientifically interpreted this phenomenon as the differences in fertility of and the competition between different pollen grains used. Unsurprisingly, the law of free combination that Mendel discovered already clarified to him that all pollen grains—even in the same anther—usually have diverse inner compositions. From the perspective of modern genetics, this is the essential difference between the methods of controlled pollination and bulked pollination.

### Illustration of the behavior of a pair of hereditary factors in fertilization

Obviously, Mendel’s controlled pollination experiment differed considerably from that of Naudin. The latter was only a study on fertilization of plant sexual reproduction, so the resultant data were the end of his exploration. In contrast, the identified one-to-one relationship between bisexual gametes reported in the eighth letter to Nageli had been used as the basis for the further pedigree analysis of the experiments introduced in the ninth letter. Of course, this was just a classical genetics method initially created by Mendel himself. In particular, the mating system of only one fertilized ovule in one ovary yielding one seed in one fruit of *M. jalapa* provided a perfect platform for Mendel to respectively check the mutual relationships between one gamete (one pollen cell or one egg cell), one factor, and one trait. Indeed, the statement that “both (egg cells and pollen cells) are equipped with the material for creating complete identical individuals” is always self-evidently treated as Mendel’s default expression in modern genetics that *a factor residing in a gamete could determine a corresponding trait*. Clearly, this is a required underlying basis when executing pedigree analysis in a bulked pollination experiment. Just as innocently illustrated in Fig. 3a, there was always no experimental evidence for the grey box until it was later illuminated by Mendel’s *M. jalapa* work. In the pedigree of Crimson × Yellow (Fig. 3b), a pair of hereditary factors (*R* & *r*) were unambiguously positioned in the two bisexual gametes, which was Mendel’s ground breaking achievement in his single pollen grain experiment. Next, the independence between two factors separately carried by the two pollen grains was predicted and confidently held when Mendel anticipated the result of the experiment. He stated “Darwin and Virchow pointed to the high degree of independence that is typical for individual characters and whole groups of characters in animals and plants, the behavior of plant hybrids indisputably furnishes an important proof of the correctness of this point of view” (the 9th letter) [5]. Therefore, he believed that the “third hybrid color” would not emerge in the following experiments at all (Fig. S1b).

The theory of hereditary factors discovered and always held by Mendel was quite beyond his time. Most nineteenth century biologists, including Darwin and Naudin, realized there might be certain internal substances with the potential to determine organismal traits. It was Mendel who first succeeded in physically positioning a pair of hereditary factors in a pair of bisexual gametes according to their parallel behavior in fertilization. Later, Sutton, based on his observation of the behaviors of a pair of alleles in parallel to a pair of homologous chromosomes in meiosis, posited that genes occur on chromosomes, as is summarized by the chromosome theory of inheritance [29]. Analogically, Mendel’s theory of hereditary factors could be called the gamete theory of inheritance. Mendel might have confidently conceived of the gamete theory in his brain because he always used the symbols *A* and *a* to denote a pair of gamete as well as a pair of hereditary factors, implying he was sure that one gamete should carry one factor in his conception system. Sure, the gamete theory of inheritance we proposed here should be on the basis of the later-achieved knowledge of double fertilization in angiosperms. The most important step of double fertilization in angiosperms is that of one spermatozoon carrying a copy of a genome and fusing with one egg cell carrying another copy of the genome to form a diploid zygote. Indeed, the double fertilization phenomenon was first observed by the Russian biologist Sergei Nawaschin [30], almost 30 years later.

### Exemplification of the hereditary factor function in determining development of seeds and plants

**Fig. 4:**
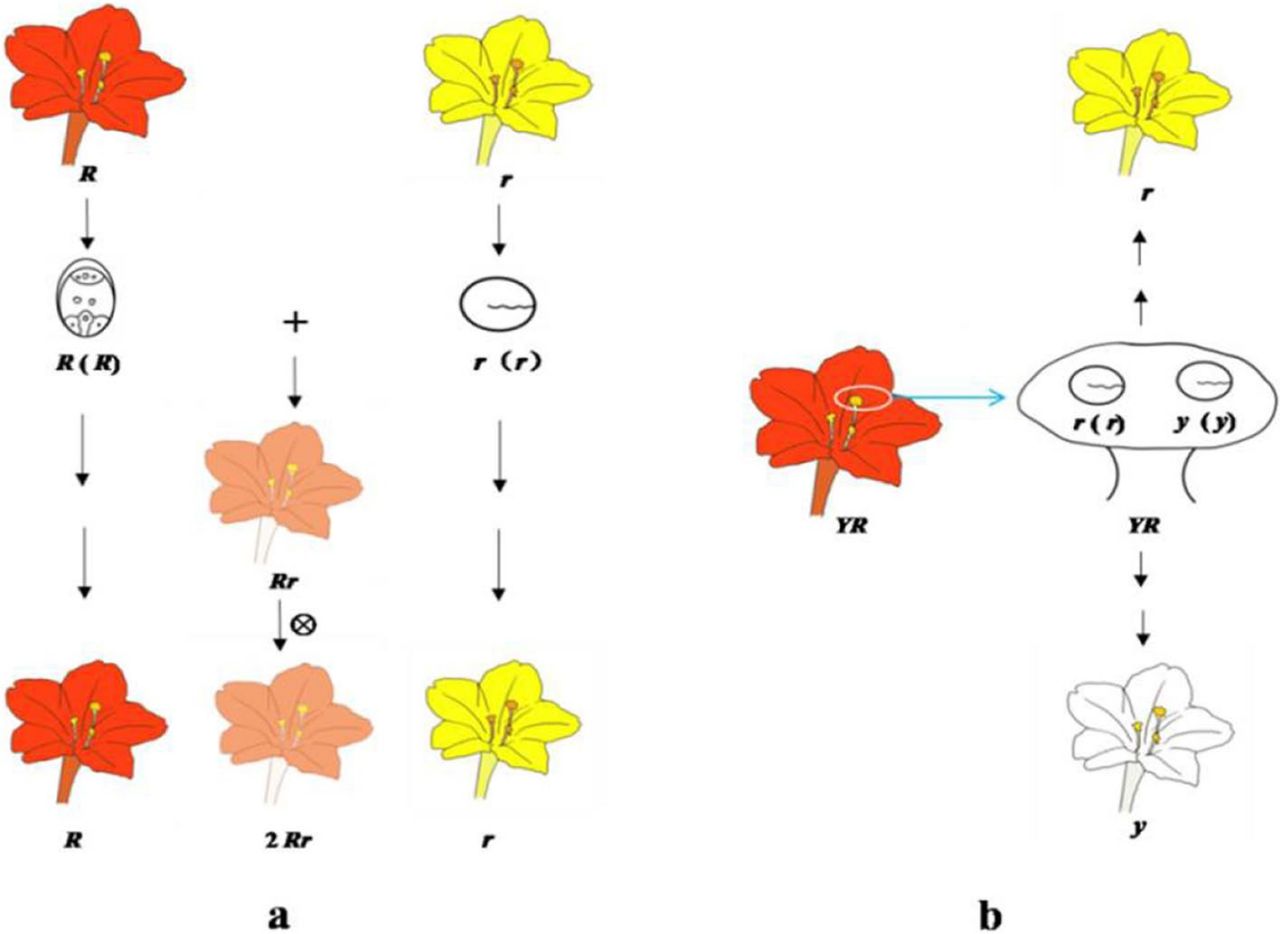
Corresponding relationships between one trait, one gamete, and one factor in the reported single pollen experiment (**a**) and in the designed two pollen experiment (**b**)

Mendel’s experiment in *M. jalapa* was also a valuable opportunity to perfectly elucidate his thoughts through choosing a pollen grain equipped with a hereditary factor to control the corresponding trait of the offspring. Mendel’s pedigree analysis of his Crimson × Yellow experiment (Fig. 3b) could be narrated by using his symbol system as follows: an egg cell *R* from a crimson flower *R* fertilizing a pollen grain *r* from a yellow flower *r* could, based on their respective hereditary factors *R* and *r*, transmit the flower colors of crimson *R* and yellow *r* to the offspring of hybrid (Fig. 4a). Hartl and Orel already noticed that in Mendel’s own conceptual system a pair of capital and lowercase letters *A* and *a* could represent different senses corresponding to different situations, i.e., a pair of contrasting traits and a pair of hereditary factors, respectively [31]. This is another piece of evidence that both letters also referred to two bisexual gametes as its third sense. Thus, Mendel’s description of his finished single pollen grain experiment could exemplify very well that *a factor residing in a gamete could determine a corresponding trait through sexual reproduction*. Similarly, in his design of the two-grain experiment (Fig. S2), after placing two pollen grains *r* and *y* respectively equipped with two hereditary factors *r* and *y* to one stigma, the observed detectable reappearance of two flower traits of yellow *r* and white color *y* in the offspring of the hybrid is anticipated (Fig. 4b).

The most unmistakable elucidation of Mendel’s theory of hereditary factors is also found in his ninth letter to Nageli, in which he explained the sex determination of *Lychnis diurnal*. “The anlage for the functional development of either the pistil alone or of the anthers alone, must have been expressed in the organization of the primordial cells from which the plants developed, and that this difference in the primordial cells could possibly be due to the ovules as well as the pollen cells being different as regards the sex anlage” (the 9th letter) [5]. Certainly, Mendel’s controlled pollination experiment in *Mirabilis* was the first case in which a single pollen grain—as a carrier of a hereditary factor—was used by man to transmit a gene to a descendent generation in order to control the corresponding trait in the offspring. In support of Mendel’s gamete theory of inheritance, this was a perfect experimental evidence as well as a wise application of the hereditary factor function for determining the corresponding trait in offspring.

### Joint construction of holistic Mendelism

The spontaneous initiation of inter-specific and intra-specific hybridization experiments in *Mirabilis* during his *Hieracium* study period also confirmed the observation that “both variable and constant hybrids were of interest to Mendel with respect to inheritance and to species evolution” [3]. Mendel highly evaluated his findings regarding variable hybrids in *Pisum*, because he believed that “in important points a fundamental difference cannot occur, because the unity in the developmental plan of organic life is beyond question” [9]. Here, Mendel casted more emphasis on the intra-specific experiments in *M. jalapa* rather than the inter-specific experiments in *Mirabilis*, which also revealed that Mendel never forgot his initial aim to explore the inheritance principle of variable hybrids. After the disturbance of Mendel’s speculation about constant hybrids in his paper were disregarded, the modified perfect formula, *A*/*A* + *a*/*a* + *A*/*a* + *a*/*A* = *A* + 2*Aa* + *a*, could indeed represent his core discovery regarding variable hybrids. That was also clearly presented via early lecture as the hypothesis of hereditary factors regarding *plant sexual reproduction in general despite hybridization particularly*, so the reproduction of homozygotes must be the integral part of his theory as a whole. As listed above, Mendel presented the union of two like elements as just “*A*/*A*” and “*a*/*a*” for dominance and recessiveness, respectively, both of which were mutually separating but not blending.

**Fig. 5:**
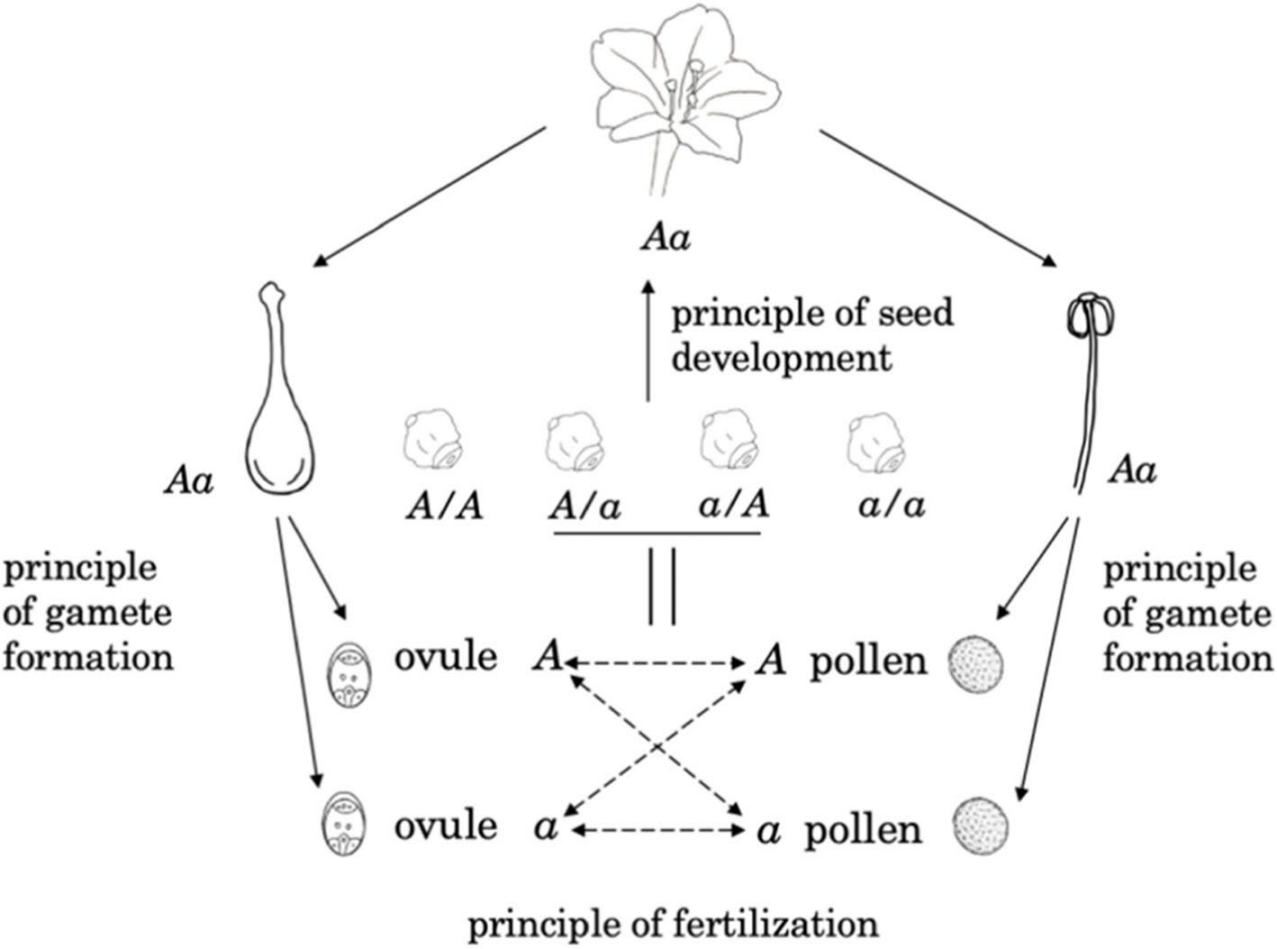
Reconstruction of Mendel’s three modules of “principles regarding gamete formation, fertilization, and seed development in general and in hybrids in particular”

Olby defined “a Mendelian as one who subscribes explicitly to the existence of a finite number of hereditary elements which in the simplest case is two per hereditary trait, only one of which may enter one germ cell” [17]. So far, the holistic Mendelism—orally presented as the hereditary factor hypothesis containing the principles regarding gamete formation, fertilization, and seed development—could be illustrated in Fig. 5, which demonstrates flower color inheritance in *M. jalapa*. Obviously, the principle of gamete formation was previously demonstrated in *Pisum* by using bulked pollination experiments in testcrosses, while the principle of fertilization and of seed development were later confirmed in his controlled pollination experiments in *M. jalapa*.

## Conclusions

The publication of Mendel’s paper in 1866 was a link connecting his preceding discovery of inheritance regarding variable hybrids and his following initiation of study on constant hybrids. In the context of intense interest in hybrid species, Mendel’s reluctant accommodation of the two incompatible topics into one publication resulted in the core findings of his theory of hereditary factors being weakened and muddled to some degree. Consequently, different readers could draw different conclusions from the same publication. Fortunately, the subtle clue from his lecture regarding the hypothesis of hereditary factors was recorded in the newly accessible newspaper, and also illustrated by his controlled pollination experiment in *M. jalapa*. It was rather fortunate that the same clue about Mendel’s resultant pedigree from the single pollen grain experiment and just an experimental design of the two grain pollination experiment were contained in the surviving ninth letter to Nageli.

To disprove Darwin’s citation of Naudin’s work, Mendel immediately conducted his *M. jalapa* experiment to remedy the deficiency in his prior discovery. Through changing the operation target from a group of grains to a single pollen grain, Mendel succeeded in confirming the referenced opinion of one pollen grain uniting with one egg in fertilization. Further, his detailed pedigree introduction of the resultant single pollen grain and the designed two pollen grain experiments demonstrated that one hereditary factor carried by one gamete could independently transmit a trait to offspring through its seed development and then plant development. Thus, the leaving question regarding the internal composition of gametes in variable hybrids was solved to some extent. Finally, together with the already testified principle of gamete formation that included the law of segregation and of free combination in *Pisum*, Mendel succeeded in integrating the later validated principles of fertilization and of seed development in *M. jalapa* into a holistic Mendelism. Here it is also coined as Mendel’s gamete theory of inheritance, for awarding his creative observations of one factor carried by one gamete.

No matter how broad the scope of research interest Mendel possessed in his career, his role as the founder of genetics is becoming clearer and clearer. Here, the recovered experiments in *M. jalapa* together with the revised expression of his finding in *Pisum* provide evidence that can resolve many historical controversies regarding his contributions to genetics.

## Supplementary Information

**Additional file 1.**

## Data Availability

All data are presented in Additional file 1.

## Abbreviation

NSSB: Natural Science Society of Brno
