# Supplementary material for: Mendel’s controlled pollination experiments in Mirabilis jalapa confirmed his discovery of the gamete theory of inheritance in Pisum
Source: Hereditas. 2022 Mar 26;159:19. doi: 10.1186/s41065-022-00232-1 (PMC8961923; doi:10.1186/s41065-022-00232-1)
Supplement: Supplementary file 1 — Additional file 1. [file 41065_2022_232_MOESM1_ESM.pdf]

Mendel's Controlled Pollination Experiments in *Mirabilis jalapa* Confirmed His Discovery of the Gamete Theory of Inheritance in *Pisum*

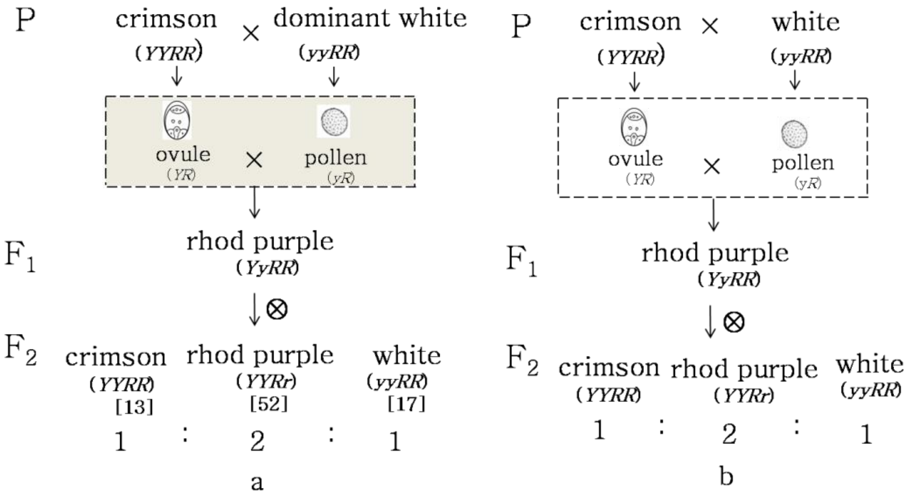

Figure S1 Putative pedigrees of Crimson × white in *Mirabilis jalapa*. **a**, pedigree adopted from Showalter (1934), the gray square indicates imprecise relationship between pollens and eggs in bulked pollination before Mendel's *M. jalapa* work; b. assumed pedigree of the single pollen experiment completed by Mendel; the rectangle marks Mendel's precise operation between one pollen grain and one ovule in *M. jalapa.*

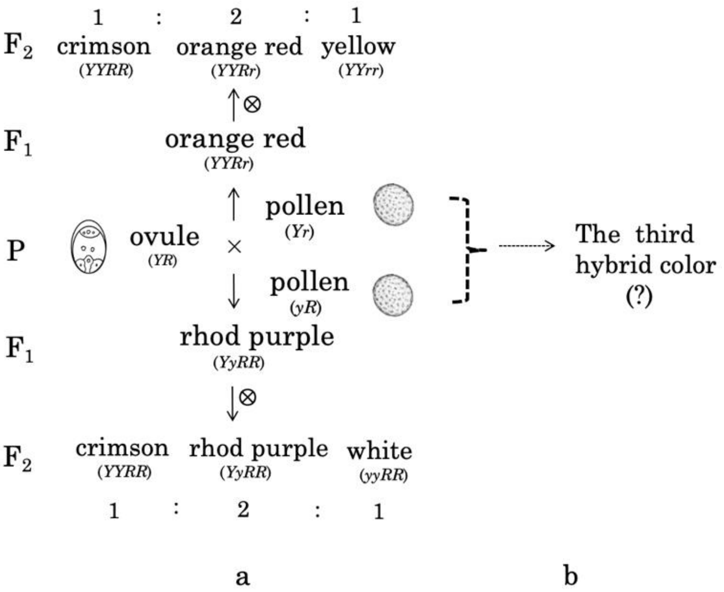

Figure S2 Putative pedigrees of double pollen pollinations designed by Mendel (a) and its alternative negative result proposed by Mendel (b)
